# A44 PGC1-α INHIBITION WORSENS DSS-INDUCED COLITIS IN MICE

**DOI:** 10.1093/jcag/gwab049.043

**Published:** 2022-02-21

**Authors:** J P Oliveira Ponce, F Lopes

**Affiliations:** Anatomy and Cell Biology, McGill University, Montreal, QC, Canada

## Abstract

**Background:**

Inflammatory bowel diseases (IBD) represent a wide range of gastrointestinal diseases that are characterized by chronic inflammation of the gastrointestinal tract (GIT). Affecting more than 6 million individuals globally, the most prevalent forms of IBD are Ulcerative colitis (UC), characterized by inflammation and ulceration of the colon and rectum, and Crohn’s disease (CD), characterized by inflammation of several areas of the GIT. Due to the morbidity and occasionally mortality arising from IBD, as well as its increasing global prevalence, the search for novel treatments and cures for IBD presents great importance. Loss of immune tolerance significantly contributes to the inflammatory patterns observed in IBD patients. Dendritic Cells (DCs), key antigen-presenting cells, are important mediators of tolerance in the gut when polarized to tolerogenic DCs (tolDCs). The phenotype of tolDCs has been shown to directly depend on their intracellular metabolism, mostly by adopting mitochondrial oxidative phosphorylation. Peroxisome proliferator-activated receptor-gamma coactivator (PGC)1-α is a transcription coactivator that plays a crucial role in mediating cellular energy metabolism. Evidence shows that activation of PGC1-α stimulates mitochondrial biogenesis and promotes oxidative metabolism in a variety of biological processes.

**Aims:**

The aim of this project was to evaluate whether activation or inhibition of PGC1-α impacts inflammatory response in DSS-induced colitic in mice. We hypothesize that the PGC1-α pharmacological activator ZLN005 would ameliorate colitis as a result of tolDC polarization. On other hand, the PGC1-α pharmacological inhibitor SR18292 would increase inflammatory responses and decrease tolerance.

**Methods:**

C57/b6 mice received DSS in drinking water for 5 days, followed by 3 days of tap water. Mice were treated with vehicle, ZLN005 (10 mg/kg), or SR18292 treatment (25 mg/kg) for the 3 days of drinking tap water. Under necropsy, colitis was assessed by disease activity score (DAS), colon length, weight change, myeloperoxidase (MPO) activity assay, histopathology score, and cytokines by ELISA. Intestinal tolerance was accessed by evaluating T cells polarization to Treg, TH1, TH2, or TH17 by qPCR of the transcription factors Foxp3 (Treg), Tbx21 (TH1), Gata3 (TH2), and Rorγt (TH17) expressed in the colon.

**Results:**

Our results demonstrated that ZLN005 did not alter the inflammatory response induced by DSS, gauged by MPO assay, DAS, and histopathology, suggesting that ZLN005 was ineffective in polarizing tolDCs. However, we observed a worsening of inflammatory conditions with treatment with SR18292 with a decrease in IL-10 levels and increased MPO values, potentially stemming from the loss of tolDCs and intestinal tolerance.

**Conclusions:**

PGC1-α inhibition worsens inflammatory response in DSS-induced colitis potentially by inhibiting tolDC polarization and loss of intestinal tolerance.

**Funding Agencies:**

None

